# Grassroots Approaches To Community-Based Representative Recruitment In Multidomain Trials

**DOI:** 10.1093/geroni/igab046.260

**Published:** 2021-12-17

**Authors:** Marcus Hill

**Affiliations:** Division of Gerontology and Geriatric Medicine, Winston-Salem, North Carolina, United States

## Abstract

U.S. POINTER is a randomized, controlled, multi-domain clinical trial to slow the progression of cognitive decline within the American population via tailored and culturally-appropriate healthy lifestyle interventions. For findings to be broadly relevant across the American population, incorporating an inclusive and robust recruitment effort has been essential to form a diverse and properly representative participant cohort. As such, the trial’s inclusive enrollment goal is 30% from traditionally underrepresented communities that include those at elevated risk for Alzheimer’s disease and related disorders. To accomplish this goal, U.S. POINTER developed and deployed a grassroots recruitment strategy in partnership with outreach specialists at each site that includes a mix of evidence-based and innovative community engagement approaches. While the COVID-19 pandemic continues to present unique challenges for recruitment, our team has been able to strategize ways to continue working within the community to support trial recruitment. An overview of these methods will be presented.

